# Supplementary material for: Roles of Nutrient Limitation on Western Lake Erie CyanoHAB Toxin Production
Source: Toxins (Basel). 2021 Jan 9;13(1):47. doi: 10.3390/toxins13010047 (PMC7828104; doi:10.3390/toxins13010047)
Supplement: Supplementary file 1 [file toxins-13-00047-s001.pdf]

Supplementary Materials: Roles of nutrient limitation on Western Lake Erie CyanoHAB toxin production

Malcolm A. Barnard, Justin D. Chaffin, Haley E. Plaas, Gregory L. Boyer, Bofan Wei, Steven W. Wilhelm, Karen L. Rossignol, Jeremy S. Braddy, George S. Bullerjahn, Thomas B. Bridgeman, Timothy W. Davis, Jin Wei, Minsheng Bu, and Hans W. Paerl

Corresponding Authors: M.A. Barnard (malcolm.barnard@unc.edu) and H.W. Paerl (hans_paerl@unc.edu)

Table of Contents

Table S1. Chlorophyll a data from the experiment. S2

Table S2. Chlorophyll a production rates S5

Table S3. Microcystin data from the experiment. S7

Table S4. Biomass-normalized microcystin data from the experiment. S9

Table S5. Microcystin production rates........ S11

Table S6. Biomass-normalized microcystin production rates........ S12

Table S7. Anatoxin data from the experiment. S13

Table S8. Biomass-normalized anatoxin data from the experiment. S15

Table S9. Anatoxin production rates. S17

Table S10. Biomass-normalized anatoxin production rates. S18

Table S11. GPS coordinates of the sampling locations and incubation S20

Table S12. Compounds compromising the major ion solution used in this experiment. S20

Table S1. Chlorophyll a data from the experiment.

Sampling Period	Bay	Treatment	T0 (μg L^{-1} chl)			T1 (μg L^{-1} chl)			T2 (μg L^{-1} chl)			T3 (μg L^{-1} chl)		
June 2019	Maumee	No Dilution	12.48	±	2.91	31.78	±	2.92	83.56	±	7.93	49.58	±	9.11
	Bay	Control	n	=	3	n	=	3	n	=	3	n	=	3
June 2019	Maumee	No Dilution	12.16	±	0.85	34.58	±	2.66	81.48	±	8.15	47.78	±	7.98
	Bay	NO_3	n	=	3	n	=	3	n	=	3	n	=	3
June 2019	Maumee	No Dilution	11.54	±	0.45	30.92	±	3.17	80.46	±	9.88	58.34	±	7.85
	Bay	NH_4	n	=	3	n	=	3	n	=	3	n	=	3
June 2019	Maumee	No Dilution	12.35	±	0.64	30.39	±	2.46	105.97	±	2.33	155.07	±	8.64
	Bay	PO_4	n	=	3	n	=	3	n	=	3	n	=	3
June 2019	Maumee	No Dilution	12.33	±	1.49	36.36	±	1.82	105.78	±	12.88	169.75	±	3.47
	Bay	N+P	n	=	3	n	=	3	n	=	3	n	=	3
June 2019	Maumee	40% Dilution	7.70	±	0.56	21.63	±	1.80	59.68	±	16.04	40.52	±	2.10
	Bay	Control	n	=	3	n	=	3	n	=	3	n	=	3
June 2019	Maumee	40% Dilution	7.41	±	0.37	20.77	±	3.27	33.97	±	4.43	27.89	±	3.83
	Bay	NO_3	n	=	3	n	=	3	n	=	3	n	=	3
June 2019	Maumee	40% Dilution	7.60	±	0.23	21.09	±	0.59	51.94	±	1.58	37.55	±	1.87
	Bay	NH_4	n	=	3	n	=	3	n	=	3	n	=	3
June 2019	Maumee	40% Dilution	7.69	±	0.20	19.46	±	0.98	67.41	±	7.41	75.74	±	3.37
	Bay	PO_4	n	=	3	n	=	3	n	=	3	n	=	3
June 2019	Maumee	40% Dilution	7.00	±	0.80	20.18	±	2.44	76.12	±	4.40	91.89	±	4.96
	Bay	N+P	n	=	3	n	=	3	n	=	3	n	=	3
June 2019	Sandusky	No Dilution	31.75	±	0.56	30.29	±	2.04	44.02	±	3.55	66.68	±	3.36
	Bay	Control	n	=	3	n	=	3	n	=	3	n	=	3
June 2019	Sandusky	No Dilution	31.40	±	0.94	29.73	±	0.92	40.76	±	4.74	63.18	±	3.40
	Bay	NO_3	n	=	3	n	=	3	n	=	3	n	=	3
June 2019	Sandusky	No Dilution	31.26	±	1.40	31.60	±	1.55	40.61	±	2.63	64.25	±	3.96
	Bay	NH_4	n	=	3	n	=	3	n	=	3	n	=	3
June 2019	Sandusky	No Dilution	31.26	±	0.39	30.89	±	1.30	43.62	±	5.14	68.87	±	8.57
	Bay	PO_4	n	=	3	n	=	3	n	=	3	n	=	3
June 2019	Sandusky	No Dilution	21.79	±	1.24	19.69	±	4.04	41.07	±	2.22	74.52	±	2.16
	Bay	N+P	n	=	3	n	=	3	n	=	3	n	=	3

Sampling Period	Bay	Treatment	T0 (μg L^{-1} chl)			T1 (μg L^{-1} chl)			T2 (μg L^{-1} chl)			T3 (μg L^{-1} chl)		
June 2019	Sandusky	40% Dilution	18.04	±	0.65	17.75	±	0.89	22.25	±	1.75	33.18	±	2.63
	Bay	Control	n	=	3	n	=	3	n	=	3	n	=	3
June 2019	Sandusky	40% Dilution	19.12	±	0.40	18.39	±	0.51	23.78	±	0.36	45.75	±	18.66
	Bay	NO_3	n	=	3	n	=	3	n	=	3	n	=	3
June 2019	Sandusky	40% Dilution	17.31	±	1.12	17.32	±	1.01	23.41	±	2.85	33.64	±	3.36
	Bay	NH_4	n	=	3	n	=	3	n	=	3	n	=	3
June 2019	Sandusky	40% Dilution	18.25	±	0.84	17.14	±	1.13	23.12	±	2.88	36.12	±	2.44
	Bay	PO_4	n	=	3	n	=	3	n	=	3	n	=	3
June 2019	Sandusky	40% Dilution	19.11	±	0.35	18.59	±	1.00	22.77	±	1.10	35.23	±	2.92
	Bay	N+P	n	=	3	n	=	3	n	=	3	n	=	3
August 2019	Maumee	No Dilution	428.40	±	35.47	331.03	±	18.83	215.08	±	31.47	97.00	±	14.83
	Bay	Control	n	=	3	n	=	3	n	=	3	n	=	3
August 2019	Maumee	No Dilution	408.92	±	28.16	281.70	±	17.90	196.15	±	25.11	77.13	±	9.90
	Bay	NO_3	n	=	3	n	=	3	n	=	3	n	=	3
August 2019	Maumee	No Dilution	390.22	±	41.66	345.26	±	36.80	200.58	±	44.67	85.50	±	17.12
	Bay	NH_4	n	=	3	n	=	3	n	=	3	n	=	3
August 2019	Maumee	No Dilution	378.22	±	12.08	318.07	±	14.73	162.84	±	30.16	70.23	±	11.51
	Bay	Urea	n	=	3	n	=	3	n	=	3	n	=	3
August 2019	Maumee	No Dilution	391.93	±	9.16	331.18	±	1.21	184.85	±	12.19	95.40	±	16.05
	Bay	PO_4	n	=	3	n	=	3	n	=	3	n	=	3
August 2019	Maumee	No Dilution	390.88	±	11.18	354.75	±	15.11	217.65	±	11.40	107.31	±	9.99
	Bay	N+P	n	=	3	n	=	3	n	=	3	n	=	3
August 2019	Maumee	40% Dilution	228.64	±	8.69	250.21	±	27.78	166.43	±	16.72	107.50	±	7.89
	Bay	Control	n	=	3	n	=	3	n	=	3	n	=	3
August 2019	Maumee	40% Dilution	218.77	±	2.69	244.57	±	14.99	151.19	±	5.70	70.00	±	22.02
	Bay	NO_3	n	=	3	n	=	3	n	=	3	n	=	3
August 2019	Maumee	40% Dilution	233.67	±	2.71	224.01	±	20.44	168.98	±	6.94	87.46	±	5.52
	Bay	NH_4	n	=	3	n	=	3	n	=	3	n	=	3
August 2019	Maumee	40% Dilution	241.33	±	0.89	215.56	±	3.39	137.88	±	24.86	50.51	±	7.45
	Bay	Urea	n	=	3	n	=	3	n	=	3	n	=	3
August 2019	Maumee	40% Dilution	216.82	±	3.80	214.56	±	6.75	160.87	±	7.27	102.51	±	14.06
	Bay	PO_4	n	=	3	n	=	3	n	=	3	n	=	3

**Sampling Period**	**Bay**	**Treatment**	**T0 (μg L⁻¹ chl)**			**T1 (μg L⁻¹ chl)**			**T2 (μg L⁻¹ chl)**			**T3 (μg L⁻¹ chl)**		
August 2019	Maumee Bay	40% Dilution N+P	235.67	±	6.27	213.25	±	9.67	143.30	±	1.21	66.89	±	17.30
			n	=	3	n	=	3	n	=	3	n	=	3
August 2019	Sandusky Bay	No Dilution Control	63.81	±	1.00	44.99	±	1.32	32.69	±	3.55	29.96	±	3.20
			n	=	3	n	=	3	n	=	3	n	=	3
August 2019	Sandusky Bay	No Dilution NO_3	92.11	±	2.29	113.46	±	1.76	124.01	±	7.72	137.10	±	10.64
			n	=	3	n	=	3	n	=	3	n	=	3
August 2019	Sandusky Bay	No Dilution NH_4	85.46	±	9.44	108.16	±	3.09	117.84	±	1.84	127.44	±	4.86
			n	=	3	n	=	3	n	=	3	n	=	3
August 2019	Sandusky Bay	No Dilution Urea	75.52	±	2.85	108.91	±	4.33	116.21	±	3.21	126.68	±	5.27
			n	=	3	n	=	3	n	=	3	n	=	3
August 2019	Sandusky Bay	No Dilution PO_4	61.46	±	1.27	43.44	±	2.76	36.26	±	3.06	36.59	±	0.70
			n	=	3	n	=	3	n	=	3	n	=	3
August 2019	Sandusky Bay	No Dilution N+P	77.78	±	3.70	127.40	±	5.05	155.39	±	3.33	196.34	±	3.19
			n	=	3	n	=	3	n	=	3	n	=	3
August 2019	Sandusky Bay	40% Dilution Control	46.10	±	3.08	33.40	±	1.32	63.16	±	22.36	23.97	±	4.53
			n	=	3	n	=	3	n	=	3	n	=	3
August 2019	Sandusky Bay	40% Dilution NO_3	45.99	±	1.89	72.12	±	4.59	62.34	±	30.16	87.07	±	1.46
			n	=	2	n	=	3	n	=	3	n	=	3
August 2019	Sandusky Bay	40% Dilution NH_4	46.07	±	0.91	64.20	±	11.87	68.68	±	6.61	79.78	±	4.33
			n	=	3	n	=	3	n	=	3	n	=	3
August 2019	Sandusky Bay	40% Dilution Urea	49.65	±	2.78	70.26	±	0.94	66.61	±	9.37	86.86	±	2.27
			n	=	3	n	=	3	n	=	3	n	=	3
August 2019	Sandusky Bay	40% Dilution PO_4	46.13	±	3.11	30.82	±	1.57	23.21	±	1.86	25.62	±	1.50
			n	=	3	n	=	3	n	=	3	n	=	3
August 2019	Sandusky Bay	40% Dilution N+P	46.84	±	1.93	80.57	±	6.88	160.05	±	9.22	169.26	±	2.78
			n	=	3	n	=	3	n	=	3	n	=	3

Table S2. Chlorophyll a production rates.

Sampling Period	Bay	Treatment	Production (d^{-1})		
June 2019	Maumee Bay	No Dilution Control	0.46	±	0.34
June 2019	Maumee Bay	No Dilution NO_3	0.46	±	0.19
June 2019	Maumee Bay	No Dilution NH_4	0.54	±	0.14
June 2019	Maumee Bay	No Dilution PO_4	0.84	±	0.08
June 2019	Maumee Bay	No Dilution N+P	0.87	±	0.15
June 2019	Maumee Bay	40% Dilution Control	0.55	±	0.10
June 2019	Maumee Bay	40% Dilution NO_3	0.44	±	0.15
June 2019	Maumee Bay	40% Dilution NH_4	0.53	±	0.06
June 2019	Maumee Bay	40% Dilution PO_4	0.76	±	0.05
June 2019	Maumee Bay	40% Dilution N+P	0.86	±	0.15
June 2019	Sandusky Bay	No Dilution Control	0.25	±	0.05
June 2019	Sandusky Bay	No Dilution NO_3	0.23	±	0.07
June 2019	Sandusky Bay	No Dilution NH_4	0.25	±	0.08
June 2019	Sandusky Bay	No Dilution PO_4	0.25	±	0.13
June 2019	Sandusky Bay	No Dilution N+P	0.30	±	0.06
June 2019	Sandusky Bay	40% Dilution Control	0.21	±	0.09
June 2019	Sandusky Bay	40% Dilution NO_3	0.30	±	0.41
June 2019	Sandusky Bay	40% Dilution NH_4	0.20	±	0.12
June 2019	Sandusky Bay	40% Dilution PO_4	0.24	±	0.09
June 2019	Sandusky Bay	40% Dilution N+P	0.21	±	0.09
August 2019	Maumee Bay	No Dilution Control	-0.50	±	0.18
August 2019	Maumee Bay	No Dilution NO_3	-0.56	±	0.15
August 2019	Maumee Bay	No Dilution NH_4	-0.51	±	0.24
August 2019	Maumee Bay	No Dilution Urea	-0.56	±	0.17

**Sampling Period**	**Bay**	**Treatment**	**Production (d^{-1})**		
August 2019	Maumee Bay	No Dilution PO_4	-0.47	±	0.17
August 2019	Maumee Bay	No Dilution N+P	-0.43	±	0.10
August 2019	Maumee Bay	40% Dilution Control	-0.25	±	0.09
August 2019	Maumee Bay	40% Dilution NO_3	-0.38	±	0.31
August 2019	Maumee Bay	40% Dilution NH_4	-0.33	±	0.06
August 2019	Maumee Bay	40% Dilution Urea	-0.52	±	0.15
August 2019	Maumee Bay	40% Dilution PO_4	-0.25	±	0.14
August 2019	Maumee Bay	40% Dilution N+P	-0.42	±	0.26
August 2019	Sandusky Bay	No Dilution Control	-0.25	±	0.11
August 2019	Sandusky Bay	No Dilution NO_3	0.13	±	0.08
August 2019	Sandusky Bay	No Dilution NH_4	0.13	±	0.14
August 2019	Sandusky Bay	No Dilution Urea	0.17	±	0.06
August 2019	Sandusky Bay	No Dilution PO_4	-0.17	±	0.03
August 2019	Sandusky Bay	No Dilution N+P	0.31	±	0.06
August 2019	Sandusky Bay	40% Dilution Control	-0.22	±	0.21
August 2019	Sandusky Bay	40% Dilution NO_3	0.21	±	0.05
August 2019	Sandusky Bay	40% Dilution NH_4	0.18	±	0.06
August 2019	Sandusky Bay	40% Dilution Urea	0.19	±	0.07
August 2019	Sandusky Bay	40% Dilution PO_4	-0.20	±	0.10
August 2019	Sandusky Bay	40% Dilution N+P	0.43	±	0.05

Table S3. Microcystin data from the experiment.

Sampling Period	Bay	Treatment	T0 (μg L^{-1})	T1 (μg L^{-1})	T2 (μg L^{-1})	T3 (μg L^{-1})
June 2019	Sandusky Bay	No Dilution<br>Control	0.14 ± 0.03<br>n = 3	0.17 ± 0.02<br>n = 3	0.20 ± 0.05<br>n = 3	0.29 ± 0.05<br>n = 3
June 2019	Sandusky Bay	No Dilution<br>NO_3	0.17 ± 0.01<br>n = 2	0.18 ± 0.04<br>n = 3	0.25 ± 0.04<br>n = 3	0.29 ± 0.07<br>n = 3
June 2019	Sandusky Bay	No Dilution<br>NH_4	0.18 ± 0.01<br>n = 3	0.19 ± 0.03<br>n = 3	0.23 ± 0.02<br>n = 3	0.29 ± 0.03<br>n = 3
June 2019	Sandusky Bay	No Dilution<br>PO_4	0.23 ± 0.02<br>n = 3	0.25 ± 0.05<br>n = 3	0.20 ± 0.02<br>n = 3	0.36 ± 0.01<br>n = 3
June 2019	Sandusky Bay	No Dilution<br>N+P	0.20 ± 0.04<br>n = 2	0.19 ± 0.03<br>n = 3	0.23 ± 0.02<br>n = 3	0.35 ± 0.04<br>n = 3
June 2019	Sandusky Bay	40% Dilution<br>Control	0.14 ± 0.02<br>n = 2	0.13 ± 0.06<br>n = 2	0.12 ± 0.01<br>n = 2	0.15 ± 0.06<br>n = 2
June 2019	Sandusky Bay	40% Dilution<br>NO_3	0.18 ± 0.06<br>n = 2	0.11 ± 0.01<br>n = 3	0.13 ± 0.04<br>n = 3	0.14 ± 0.01<br>n = 2
June 2019	Sandusky Bay	40% Dilution<br>NH_4	0.14 ± 0.00<br>n = 1	0.12 ± 0.03<br>n = 3	0.15 ± 0.03<br>n = 3	0.12 ± 0.05<br>n = 3
June 2019	Sandusky Bay	40% Dilution<br>PO_4	0.23 ± 0.00<br>n = 1	0.11 ± 0.03<br>n = 3	0.13 ± 0.01<br>n = 2	0.15 ± 0.04<br>n = 2
June 2019	Sandusky Bay	40% Dilution<br>N+P	0.16 ± 0.00<br>n = 1	0.14 ± 0.02<br>n = 3	0.14 ± 0.03<br>n = 3	0.13 ± 0.01<br>n = 3
August 2019	Maumee Bay	No Dilution<br>Control	18.06 ± 1.10<br>n = 3	14.91 ± 1.28<br>n = 3	9.39 ± 3.27<br>n = 3	2.79 ± 1.13<br>n = 3
August 2019	Maumee Bay	No Dilution<br>NO_3	16.25 ± 1.01<br>n = 3	14.90 ± 2.04<br>n = 3	7.36 ± 2.22<br>n = 3	1.42 ± 0.74<br>n = 3
August 2019	Maumee Bay	No Dilution<br>NH_4	16.33 ± 0.89<br>n = 3	14.15 ± 1.65<br>n = 3	8.11 ± 3.70<br>n = 3	2.38 ± 1.11<br>n = 3
August 2019	Maumee Bay	No Dilution<br>Urea	17.49 ± 1.52<br>n = 3	16.21 ± 2.84<br>n = 3	5.66 ± 1.05<br>n = 3	2.61 ± 1.11<br>n = 3
August 2019	Maumee Bay	No Dilution<br>PO_4	17.38 ± 0.42<br>n = 3	12.22 ± 0.87<br>n = 3	6.44 ± 1.48<br>n = 3	2.82 ± 1.74<br>n = 3

**Sampling Period**	**Bay**	**Treatment**	**T0 (µg L^{-1})**			**T1 (µg L^{-1})**			**T2 (µg L^{-1})**			**T3 (µg L^{-1})**		
August 2019	Maumee Bay	No Dilution	16.75	±	1.16	11.44	±	0.33	5.09	±	0.42	3.32	±	1.19
		N+P	n	=	3	n	=	3	n	=	3	n	=	3
August 2019	Maumee Bay	40% Dilution	11.47	±	2.46	10.65	±	1.89	10.29	±	1.61	3.34	±	1.16
		Control	n	=	3	n	=	3	n	=	3	n	=	3
August 2019	Maumee Bay	40% Dilution	12.40	±	0.99	8.66	±	0.63	8.83	±	1.57	3.37	±	1.01
		NO_3	n	=	3	n	=	3	n	=	3	n	=	3
August 2019	Maumee Bay	40% Dilution	11.71	±	1.85	11.16	±	1.91	8.79	±	1.80	2.75	±	0.56
		NH_4	n	=	3	n	=	3	n	=	3	n	=	3
August 2019	Maumee Bay	40% Dilution	8.76	±	1.54	10.06	±	1.37	8.47	±	1.26	1.48	±	0.61
		Urea	n	=	3	n	=	3	n	=	3	n	=	3
August 2019	Maumee Bay	40% Dilution	8.41	±	0.87	12.12	±	1.26	8.65	±	0.64	4.57	±	1.66
		PO_4	n	=	3	n	=	3	n	=	3	n	=	3
August 2019	Maumee Bay	40% Dilution	7.76	±	1.02	11.83	±	0.23	8.20	±	0.88	3.28	±	0.66
		N+P	n	=	3	n	=	3	n	=	3	n	=	3

Table S4. Biomass-normalized microcystin data from the experiment.

Sampling Period	Bay	Treatment	T0 (µg microcystin µg chlorophyll a $^{-1}$)			T1 (µg microcystin µg chlorophyll a $^{-1}$)			T2 (µg microcystin µg chlorophyll a $^{-1}$)			T3 (µg microcystin µg chlorophyll a $^{-1}$)		
June 2019	Sandusky Bay	No Dilution	0.004	±	0.040	0.006	±	0.009	0.005	±	0.014	0.004	±	0.015
		Control	n	=	3	n	=	3	n	=	3	n	=	3
June 2019	Sandusky Bay	No Dilution	0.006	±	0.009	0.006	±	0.036	0.006	±	0.009	0.005	±	0.020
		NO_3	n	=	2	n	=	3	n	=	3	n	=	3
June 2019	Sandusky Bay	No Dilution	0.006	a	0.005	0.006	±	0.015	0.006	±	0.006	0.005	±	0.008
		NH_4	n		3	n	=	3	n	=	3	n	=	3
June 2019	Sandusky Bay	No Dilution	0.007	±	0.047	0.008	±	0.034	0.005	±	0.003	0.005	±	0.001
		PO_4	n	=	3	n	=	3	n	=	3	n	=	3
June 2019	Sandusky Bay	No Dilution	0.007	±	0.032	0.007	±	0.014	0.006	±	0.025	0.005	±	0.002
		N+P	n	=	2	n	=	3	n	=	3	n	=	3
June 2019	Sandusky Bay	40% Dilution	0.009	±	0.142	0.006	±	0.069	0.005	±	0.004	0.003	±	0.028
		Control	n	=	2	n	=	2	n	=	2	n	=	3
June 2019	Sandusky Bay	40% Dilution	0.008	±	0.034	0.007	±	0.019	0.005	±	0.110	0.005	±	0.001
		NO_3	n	=	2	n	=	3	n	=	3	n	=	2
June 2019	Sandusky Bay	40% Dilution	0.007	±	0.00	0.007	±	0.022	0.007	±	0.009	0.004	±	0.015
		NH_4	n	=	1	n	=	3	n	=	3	n	=	3
June 2019	Sandusky Bay	40% Dilution	0.013	±	0.00	0.007	±	0.022	0.006	±	0.004	0.004	±	0.021
		PO_4	n	=	1	n	=	3	n	=	2	n	=	2
June 2019	Sandusky Bay	40% Dilution	0.009	±	0.00	0.008	±	0.007	0.006	±	0.011	0.004	±	0.018
		N+P	n	=	1	n	=	3	n	=	3	n	=	3
August 2019	Maumee Bay	No Dilution	0.042	±	0.025	0.045	±	0.055	0.044	±	0.104	0.029	±	0.076
		Control	n	=	3	n	=	3	n	=	3	n	=	3
August 2019	Maumee Bay	No Dilution	0.040	±	0.029	0.053	±	0.093	0.038	±	0.088	0.018	±	0.075
		NO_3	n	=	3	n	=	3	n	=	3	n	=	3
August 2019	Maumee Bay	No Dilution	0.042	±	0.017	0.041	±	0.037	0.040	±	0.083	0.028	±	0.065
		NH_4	n	=	3	n	=	3	n	=	3	n	=	3
August 2019	Maumee Bay	No Dilution	0.046	±	0.103	0.051	±	0.157	0.035	±	0.035	0.037	±	0.097
		Urea	n	=	3	n	=	3	n	=	3	n	=	3
August 2019	Maumee Bay	No Dilution	0.044	±	0.037	0.037	±	0.588	0.035	±	0.122	0.030	±	0.108
		PO_4	n	=	3	n	=	3	n	=	3	n	=	3

Sampling Period	Bay	Treatment	T0 (µg microcystin µg chlorophyll a $^{-1}$)	T1 (µg microcystin µg chlorophyll a $^{-1}$)	T2 (µg microcystin µg chlorophyll a $^{-1}$)	T3 (µg microcystin µg chlorophyll a $^{-1}$)
August 2019	Maumee Bay	No Dilution N+P	0.043 ± 0.085 n = 3	0.032 ± 0.018 n = 3	0.023 ± 0.036 n = 3	0.031 ± 0.119 n = 3
August 2019	Maumee Bay	40% Dilution Control	0.050 ± 0.231 n = 3	0.043 ± 0.055 n = 3	0.062 ± 0.096 n = 3	0.031 ± 0.147 n = 3
August 2019	Maumee Bay	40% Dilution NO_3	0.057 ± 0.300 n = 3	0.035 ± 0.034 n = 3	0.058 ± 0.276 n = 3	0.048 ± 0.046 n = 3
August 2019	Maumee Bay	40% Dilution NH_4	0.050 ± 0.556 n = 3	0.050 ± 0.076 n = 3	0.052 ± 0.260 n = 3	0.031 ± 0.101 n = 3
August 2019	Maumee Bay	40% Dilution Urea	0.036 ± 1.415 n = 3	0.047 ± 0.330 n = 3	0.061 ± 0.051 n = 3	0.029 ± 0.082 n = 3
August 2019	Maumee Bay	40% Dilution PO_4	0.039 ± 0.186 n = 3	0.057 ± 0.153 n = 3	0.054 ± 0.088 n = 3	0.045 ± 0.118 n = 3
August 2019	Maumee Bay	40% Dilution N+P	0.033 ± 0.132 n = 3	0.055 ± 0.019 n = 3	0.057 ± 0.724 n = 3	0.049 ± 0.038 n = 3

Table S5. Microcystin production rates.

Sampling Period	Bay	Treatment	Production (d^{-1})		
June 2019	Sandusky Bay	No Dilution Control	0.252	±	0.263
June 2019	Sandusky Bay	No Dilution NO_3	0.166	±	0.240
June 2019	Sandusky Bay	No Dilution NH_4	0.170	±	0.118
June 2019	Sandusky Bay	No Dilution PO_4	0.150	±	0.102
June 2019	Sandusky Bay	No Dilution N+P	0.190	±	0.235
June 2019	Sandusky Bay	40% Dilution Control	0.024	±	0.427
June 2019	Sandusky Bay	40% Dilution NO_3	-0.075	±	0.326
June 2019	Sandusky Bay	40% Dilution NH_4	-0.050	±	0.422
June 2019	Sandusky Bay	40% Dilution PO_4	-0.140	±	0.279
June 2019	Sandusky Bay	40% Dilution N+P	-0.079	±	0.037
August 2019	Maumee Bay	No Dilution Control	-0.623	±	0.411
August 2019	Maumee Bay	No Dilution NO_3	-0.812	±	0.526
August 2019	Maumee Bay	No Dilution NH_4	-0.642	±	0.468
August 2019	Maumee Bay	No Dilution Urea	-0.634	±	0.435
August 2019	Maumee Bay	No Dilution PO_4	-0.606	±	0.618
August 2019	Maumee Bay	No Dilution N+P	-0.539	±	0.365
August 2019	Maumee Bay	40% Dilution Control	-0.411	±	0.407
August 2019	Maumee Bay	40% Dilution NO_3	-0.434	±	0.309
August 2019	Maumee Bay	40% Dilution NH_4	-0.483	±	0.256
August 2019	Maumee Bay	40% Dilution Urea	-0.592	±	0.448
August 2019	Maumee Bay	40% Dilution PO_4	-0.203	±	0.378
August 2019	Maumee Bay	40% Dilution N+P	-0.287	±	0.242

Table S6. Biomass-normalized microcystin production rates.

Sampling Period	Bay	Treatment	Production (d^{-1})		
June 2019	Sandusky Bay	No Dilution Control	0.005	±	0.043
June 2019	Sandusky Bay	No Dilution NO_3	-0.067	±	0.022
June 2019	Sandusky Bay	No Dilution NH_4	-0.084	±	0.009
June 2019	Sandusky Bay	No Dilution PO_4	-0.100	±	0.047
June 2019	Sandusky Bay	No Dilution N+P	-0.113	±	0.002
June 2019	Sandusky Bay	40% Dilution Control	-0.200	±	0.009
June 2019	Sandusky Bay	40% Dilution NO_3	-0.186	±	0.042
June 2019	Sandusky Bay	40% Dilution NH_4	-0.373	±	0.142
June 2019	Sandusky Bay	40% Dilution PO_4	-0.249	±	0.015
June 2019	Sandusky Bay	40% Dilution N+P	-0.382	±	0.021
August 2019	Maumee Bay	No Dilution Control	-0.128	±	0.081
August 2019	Maumee Bay	No Dilution NO_3	-0.256	±	0.080
August 2019	Maumee Bay	No Dilution NH_4	-0.136	±	0.067
August 2019	Maumee Bay	No Dilution Urea	-0.072	±	0.141
August 2019	Maumee Bay	No Dilution PO_4	-0.135	±	0.115
August 2019	Maumee Bay	No Dilution N+P	-0.108	±	0.146
August 2019	Maumee Bay	40% Dilution Control	-0.159	±	0.274
August 2019	Maumee Bay	40% Dilution NO_3	-0.054	±	0.303
August 2019	Maumee Bay	40% Dilution NH_4	-0.155	±	0.565
August 2019	Maumee Bay	40% Dilution Urea	-0.071	±	1.418
August 2019	Maumee Bay	40% Dilution PO_4	0.046	±	0.220
August 2019	Maumee Bay	40% Dilution N+P	0.133	±	0.138

Table S7. Anatoxin data from the experiment.

Sampling Period	Bay	Treatment	T0 ($\mu g\ L^{-1}$)			T1 ($\mu g\ L^{-1}$)			T2 ($\mu g\ L^{-1}$)			T3 ($\mu g\ L^{-1}$)		
June 2019	Sandusky	No Dilution	0.05	±	0.04	0.04	±	0.01	0.08	±	0.05	0.25	±	0.06
	Bay	Control	n	=	3	n	=	3	n	=	3	n	=	3
June 2019	Sandusky	No Dilution	0.02	±	0.02	0.02	±	0.01	0.11	±	0.07	0.42	±	0.12
	Bay	NO_3	n	=	3	n	=	3	n	=	3	n	=	3
June 2019	Sandusky	No Dilution	0.02	±	0.03	0.03	±	0.01	0.07	±	0.04	0.29	±	0.08
	Bay	NH_4	n	=	2	n	=	3	n	=	3	n	=	3
June 2019	Sandusky	No Dilution	0.02	±	0.03	0.03	±	0.01	0.18	±	0.04	0.39	±	0.06
	Bay	PO_4	n	=	3	n	=	3	n	=	3	n	=	3
June 2019	Sandusky	No Dilution	0.03	±	0.04	0.04	±	0.03	0.13	±	0.09	0.46	±	0.17
	Bay	N+P	n	=	2	n	=	2	n	=	3	n	=	3
June 2019	Sandusky	40% Dilution	0.03	±	0.03	0.01	±	0.01	0.09	±	0.06	0.22	±	0.12
	Bay	Control	n	=	2	n	=	3	n	=	2	n	=	3
June 2019	Sandusky	40% Dilution	0.02	±	0.03	0.02	±	0.02	0.05	±	0.04	0.24	±	0.02
	Bay	NO_3	n	=	2	n	=	2	n	=	3	n	=	2
June 2019	Sandusky	40% Dilution	0.02	±	0.00	0.02	±	0.01	0.06	±	0.03	0.17	±	0.01
	Bay	NH_4	n	=	1	n	=	3	n	=	3	n	=	3
June 2019	Sandusky	40% Dilution	0.02	±	0.02	0.02	±	0.00	0.04	±	0.03	0.16	±	0.04
	Bay	PO_4	n	=	3	n	=	2	n	=	3	n	=	3
June 2019	Sandusky	40% Dilution		NaN[1]		0.03	±	0.03	0.14	±	0.10	0.19	±	0.09
	Bay	N+P				n	=	3	n	=	3	n	=	3
August 2019	Sandusky	No Dilution	18.06	±	1.10	14.91	±	1.28	0.70	±	0.11	1.00	±	0.13
	Bay	Control	n	=	3	n	=	3	n	=	3	n	=	3
August 2019	Sandusky	No Dilution	16.25	±	1.01	14.90	±	2.04	4.43	±	0.07	7.99	±	0.51
	Bay	NO_3	n	=	3	n	=	3	n	=	3	n	=	3
August 2019	Sandusky	No Dilution	16.33	±	0.89	14.15	±	1.65	2.91	±	0.35	5.10	±	0.25
	Bay	NH_4	n	=	3	n	=	3	n	=	3	n	=	3
August 2019	Sandusky	No Dilution	17.49	±	1.52	16.21	±	2.84	3.78	±	0.24	5.19	±	0.21
	Bay	Urea	n	=	3	n	=	3	n	=	3	n	=	3
August 2019	Sandusky	No Dilution	17.38	±	0.42	12.22	±	0.87	0.76	±	0.08	1.01	±	0.06
	Bay	PO_4	n	=	3	n	=	3	n	=	3	n	=	3

**Sampling Period**	**Bay**	**Treatment**	**T0 ($\mu g\ L^{-1}$)**			**T1 ($\mu g\ L^{-1}$)**			**T2 ($\mu g\ L^{-1}$)**			**T3 ($\mu g\ L^{-1}$)**		
August 2019	Sandusky	No Dilution	16.75	±	1.16	11.44	±	0.33	2.19	±	0.42	2.81	±	0.29
	Bay	N+P	n	=	3	n	=	3	n	=	3	n	=	3
August 2019	Sandusky	40% Dilution	11.47	±	2.46	10.65	±	1.89	0.52	±	0.18	0.43	±	0.24
	Bay	Control	n	=	3	n	=	3	n	=	3	n	=	3
August 2019	Sandusky	40% Dilution	12.40	±	0.99	8.66	±	0.63	2.82	±	0.15	4.54	±	0.34
	Bay	NO_3	n	=	3	n	=	3	n	=	3	n	=	3
August 2019	Sandusky	40% Dilution	11.71	±	1.85	11.16	±	1.91	1.49	±	0.30	2.71	±	0.49
	Bay	NH_4	n	=	3	n	=	3	n	=	3	n	=	3
August 2019	Sandusky	40% Dilution	8.76	±	1.54	10.06	±	1.37	1.94	±	0.08	2.95	±	0.05
	Bay	Urea	n	=	3	n	=	3	n	=	3	n	=	3
August 2019	Sandusky	40% Dilution	8.41	±	0.87	12.12	±	1.26	0.59	±	0.08	0.45	±	0.19
	Bay	PO_4	n	=	3	n	=	3	n	=	3	n	=	3
August 2019	Sandusky	40% Dilution	7.76	±	1.02	11.83	±	0.23	1.44	±	0.25	2.54	±	0.90
	Bay	N+P	n	=	3	n	=	3	n	=	3	n	=	3

[1] NaN is No data available

Table S8. Biomass-normalized anatoxin data from the experiment.

Sampling Period	Bay	Treatment	T0 (µg anatoxin µg chlorophyll a $^{-1}$)	T1 (µg anatoxin µg chlorophyll a $^{-1}$)	T2 (µg anatoxin µg chlorophyll a $^{-1}$)	T3 (µg anatoxin µg chlorophyll a $^{-1}$)
June 2019	Sandusky Bay	No Dilution Control	0.002 ± 0.031 n = 3	0.001 ± 0.005 n = 3	0.002 ± 0.013 n = 3	0.004 ± 0.019 n = 3
June 2019	Sandusky Bay	No Dilution NO_3	0.001 ± 0.014 n = 3	0.001 ± 0.009 n = 3	0.003 ± 0.014 n = 3	0.007 ± 0.035 n = 3
June 2019	Sandusky Bay	No Dilution NH_4	0.001 ± 0.016 n = 2	0.001 ± 0.004 n = 3	0.002 ± 0.014 n = 3	0.005 ± 0.021 n = 3
June 2019	Sandusky Bay	No Dilution PO_4	0.001 ± 0.056 n = 3	0.001 ± 0.006 n = 3	0.004 ± 0.008 n = 3	0.006 ± 0.007 n = 3
June 2019	Sandusky Bay	No Dilution N+P	0.001 ± 0.029 n = 2	0.001 ± 0.008 n = 2	0.003 ± 0.039 n = 3	0.006 ± 0.077 n = 3
June 2019	Sandusky Bay	40% Dilution Control	0.002 ± 0.046 n = 2	0.001 ± 0.012 n = 3	0.004 ± 0.042 n = 2	0.007 ± 0.045 n = 3
June 2019	Sandusky Bay	40% Dilution NO_3	0.001 ± 0.080 n = 2	0.001 ± 0.038 n = 2	0.002 ± 0.100 n = 3	0.005 ± 0.001 n = 2
June 2019	Sandusky Bay	40% Dilution NH_4	0.001 ± 0.00 n = 1	0.001 ± 0.005 n = 3	0.002 ± 0.011 n = 3	0.005 ± 0.004 n = 3
June 2019	Sandusky Bay	40% Dilution PO_4	0.001 ± 0.023 n = 3	0.001 ± 0.003 n = 2	0.002 ± 0.009 n = 3	0.004 ± 0.018 n = 3
June 2019	Sandusky Bay	40% Dilution N+P	NaN[1]	0.002 ± 0.024 n = 3	0.006 ± 0.089 n = 3	0.005 ± 0.030 n = 3
August 2019	Sandusky Bay	No Dilution Control	0.009 ± 0.168 n = 3	0.011 ± 0.026 n = 3	0.022 ± 0.032 n = 3	0.033 ± 0.040 n = 3
August 2019	Sandusky Bay	No Dilution NO_3	0.005 ± 0.046 n = 3	0.018 ± 0.111 n = 3	0.036 ± 0.009 n = 3	0.058 ± 0.048 n = 3
August 2019	Sandusky Bay	No Dilution NH_4	0.005 ± 0.006 n = 3	0.017 ± 0.024 n = 3	0.025 ± 0.191 n = 3	0.040 ± 0.052 n = 3
August 2019	Sandusky Bay	No Dilution Urea	0.006 ± 0.014 n = 3	0.017 ± 0.028 n = 3	0.033 ± 0.074 n = 3	0.041 ± 0.041 n = 3
August 2019	Sandusky Bay	No Dilution PO_4	0.007 ± 0.011 n = 3	0.016 ± 0.019 n = 3	0.021 ± 0.027 n = 3	0.028 ± 0.080 n = 3

**Sampling Period**	**Bay**	**Treatment**	**T0 (μg anatoxin μg chlorophyll a $^{-1}$)**			**T1 (μg anatoxin μg chlorophyll a $^{-1}$)**			**T2 (μg anatoxin μg chlorophyll a $^{-1}$)**			**T3 (μg anatoxin μg chlorophyll a $^{-1}$)**		
August 2019	Sandusky	No Dilution	0.004	±	0.002	0.008	±	0.012	0.014	±	0.127	0.014	±	0.091
	Bay	N+P	n	=	3	n	=	3	n	=	3	n	=	3
August 2019	Sandusky	40% Dilution	0.005	±	0.026	0.009	±	0.025	0.008	±	0.008	0.018	±	0.052
	Bay	Control	n	=	3	n	=	3	n	=	3	n	=	3
August 2019	Sandusky	40% Dilution	0.006	±	0.012	0.011	±	0.036	0.045	±	0.005	0.052	±	0.234
	Bay	NO_3	n	=	3	n	=	3	n	=	3	n	=	3
August 2019	Sandusky	40% Dilution	0.004	±	0.025	0.010	±	0.004	0.022	±	0.046	0.034	±	0.114
	Bay	NH_4	n	=	3	n	=	3	n	=	3	n	=	3
August 2019	Sandusky	40% Dilution	0.004	±	0.045	0.012	±	0.263	0.029	±	0.009	0.034	±	0.023
	Bay	Urea	n	=	3	n	=	3	n	=	3	n	=	3
August 2019	Sandusky	40% Dilution	0.006	±	0.023	0.010	±	0.096	0.025	±	0.043	0.017	±	0.129
	Bay	PO_4	n	=	3	n	=	3	n	=	3	n	=	3
August 2019	Sandusky	40% Dilution	0.005	±	0.032	0.005	±	0.006	0.009	±	0.027	0.015	±	0.325
	Bay	N+P	n	=	3	n	=	3	n	=	3	n	=	3

[1] NaN is No data available

Table S9. Anatoxin production rates.

Sampling Period	Bay	Treatment	Production (d^{-1})		
June 2019	Sandusky Bay	No Dilution Control	0.516	±	0.473
June 2019	Sandusky Bay	No Dilution NO_3	0.985	±	0.767
June 2019	Sandusky Bay	No Dilution NH_4	0.898	±	1.175
June 2019	Sandusky Bay	No Dilution PO_4	0.970	±	1.303
June 2019	Sandusky Bay	No Dilution N+P	0.965	±	1.459
June 2019	Sandusky Bay	40% Dilution Control	0.663	±	1.107
June 2019	Sandusky Bay	40% Dilution NO_3	0.783	±	1.416
June 2019	Sandusky Bay	40% Dilution NH_4	0.800	±	0.084
June 2019	Sandusky Bay	40% Dilution PO_4	0.627	±	1.023
June 2019	Sandusky Bay	40% Dilution N+P		NaN[1]	
August 2019	Sandusky Bay	No Dilution Control	0.171	±	0.369
August 2019	Sandusky Bay	No Dilution NO_3	1.084	±	0.284
August 2019	Sandusky Bay	No Dilution NH_4	0.795	±	0.192
August 2019	Sandusky Bay	No Dilution Urea	0.800	±	0.110
August 2019	Sandusky Bay	No Dilution PO_4	0.297	±	0.070
August 2019	Sandusky Bay	No Dilution N+P	0.717	±	0.107
August 2019	Sandusky Bay	40% Dilution Control	0.204	±	0.690
August 2019	Sandusky Bay	40% Dilution NO_3	1.020	±	0.151
August 2019	Sandusky Bay	40% Dilution NH_4	0.780	±	0.255
August 2019	Sandusky Bay	40% Dilution Urea	0.683	±	0.751
August 2019	Sandusky Bay	40% Dilution PO_4	0.158	±	0.535
August 2019	Sandusky Bay	40% Dilution N+P	0.589	±	0.489

[1] NaN is No data available

Table S10. Biomass-normalized anatoxin production rates.

Sampling Period	Bay	Treatment	Production (d^{-1})		
June 2019	Sandusky Bay	No Dilution Control	0.269	±	0.036
June 2019	Sandusky Bay	No Dilution NO_3	0.752	±	0.038
June 2019	Sandusky Bay	No Dilution NH_4	0.645	±	0.027
June 2019	Sandusky Bay	No Dilution PO_4	0.721	±	0.057
June 2019	Sandusky Bay	No Dilution N+P	0.663	±	0.082
June 2019	Sandusky Bay	40% Dilution Control	0.453	±	0.064
June 2019	Sandusky Bay	40% Dilution NO_3	0.485	±	0.080
June 2019	Sandusky Bay	40% Dilution NH_4	0.601	±	0.004
June 2019	Sandusky Bay	40% Dilution PO_4	0.385	±	0.029
June 2019	Sandusky Bay	40% Dilution N+P		NaN[1]	
August 2019	Sandusky Bay	No Dilution Control	0.423	±	0.172
August 2019	Sandusky Bay	No Dilution NO_3	0.812	±	0.067
August 2019	Sandusky Bay	No Dilution NH_4	0.723	±	0.053
August 2019	Sandusky Bay	No Dilution Urea	0.628	±	0.043
August 2019	Sandusky Bay	No Dilution PO_4	0.470	±	0.081
August 2019	Sandusky Bay	No Dilution N+P	0.441	±	0.091
August 2019	Sandusky Bay	40% Dilution Control	0.422	±	0.058
August 2019	Sandusky Bay	40% Dilution NO_3	0.749	±	0.234
August 2019	Sandusky Bay	40% Dilution NH_4	0.714	±	0.117
August 2019	Sandusky Bay	40% Dilution Urea	0.703	±	0.051
August 2019	Sandusky Bay	40% Dilution PO_4	0.354	±	0.131

August 2019	Sandusky Bay	40% Dilution N+P	0.375	±	0.327

[1] NaN is No data available

Table S11. GPS coordinates of the sampling locations and incubation.

Site	Location	GPS Coordinates
Maumee Bay Sampling	Bulkhead across from University of Toledo’s Lake Erie Center, Oregon, OH, USA	41°41'26.25"N, 83°23'52.77"W
Sandusky Bay Sampling	Paper District Marina, Sandusky, OH, USA	41°27'28.00"N, 82°49'36.62"W
Sample Incubation	The Ohio State University Stone Laboratory, Put-In-Bay, OH, USA	41°39'28.58"N, 82°49'36.62"W

Table S12. Compounds compromising the major ion solution used in this experiment.

Compound	mM	mg/L
$CaCO_3$	0.35	35.00
$NaHCO_3$	0.2	16.80
$MgCl_2$	0.21	42.63
KCl	0.04	3.00
$CaSO_4$	0.45	77.40
$MgCO_3$	0.16	13.44
